# Royal Medical Benevolent College

**Published:** 1890-04-12

**Authors:** C. Holman

**Affiliations:** Treasurer of Epsom College. Reigate


### Editor's Letter-Box.

### ROYAL MEDICAL BENEVOLENT COLLEGE.

Sir,—Allow me to direct the attention of your readers to the advertisement of the biennial festival of the Royal Medical Benevolent College on April 17th, to which you have so kindly given a prominent position. Sir James Paget has consented to take the chair in the hope of aiding the fund for the support of the pensioners and foundationers of the College. The Council have each year to find not less than £4,000 to support fifty pensioners, and to clothe, feed, and educate fifty foundation scholars. Towards this amount they have but a certain income of £600 a-year. Can there be any cause more directly appealing to the medical profession, and through them to the public, than the soothing the declining years of the necessitous aged and giving a sound and thorough education to the young? We ask for no money help for the school generally, apart from the foundation. It is doing good work, which is steadily bearing fruit. The numbers are rising, but at present there is accommodation for more pupils. It is with no little pride that we can point at this moment to as many as six Epsomians upon the teaching staff of our great metropolitan hospitals. This will sufficiently stamp the excellence of our scientific teaching. We offer immense benefits to the sons of medical men, but we do away with the evils of a class school by the admixture of the sons of laymen—sons of parents outside the medical profession. Each boy is under the special care of a house-master, who is in close relation to him at all times; and he receives an education which will fit him in due course to go direct from the school to Oxford or Cambridge, to the London hospitals, or to enter the army or go into a merchant's office. It is the desire of the Council to extend the usefulness cf the school, and to increase the benefits it confers, by attracting a larger number of pupils. They now appeal with confidence to the members of the medical profession for funds to carry on the benevolent side of their great work.—I am, Sir, yours faithfully,

C. Holman,

Reigate. Treasurer of Epsom College.

